# Human T-cell leukemia virus type 1 infection among Japanese immigrants and their descendants living in Southeast Brazil: A call for preventive and control responses

**DOI:** 10.1371/journal.pntd.0009066

**Published:** 2021-02-05

**Authors:** Larissa M. Bandeira, Marco A. M. Puga, Sabrina M. S. Weis-Torres, Grazielli R. Rezende, João A. Domingos, Tayana S. O. Tanaka, Gabriela A. Cesar, Youko Nukui, Ana C. P. Vicente, Jorge Casseb, Juliana Yamashiro, Aluísio C. Segurado, Murilo O. Saito, João R. R. Pinho, Rivaldo V. Cunha, Osnei Okumoto, Silvia N. O. Uehara, Ana R. C. Motta-Castro

**Affiliations:** 1 Universidade Federal de Mato Grosso do Sul, Campo Grande, Mato Grosso do Sul, Brazil; 2 Hospital das Clínicas, Faculdade de Medicina, Universidade de São Paulo, São Paulo, São Paulo, Brazil; 3 Instituto Oswaldo Cruz, Fiocruz, Rio de Janeiro, Rio de Janeiro, Brazil; 4 Agência Mãe, São Paulo, São Paulo, Brazil; 5 Fiocruz Mato Grosso do Sul, Fundação Oswaldo Cruz, Campo Grande, Mato Grosso do Sul, Brazil; 6 Secretaria Nacional de Vigilância em Saúde SVS, Ministério da Saúde, Brasília, Distrito Federal, Brazil; Imperial College London, UNITED KINGDOM

## Abstract

Human T-cell leukemia virus type 1 (HTLV-1) has worldwide distribution and is considered endemic in southwestern Japan. HTLV-1 infection has been associated with adult T-cell leukemia/lymphoma (ATL) and HTLV-1-associated myelopathy/tropical spastic paraparesis (HAM/TSP) besides other diseases. This cross-sectional study aimed to investigate the prevalence, risk factors and molecular characterization of HTLV-1, among the world’s largest population of Japanese immigrants and their descendants outside of Japan, in São Paulo, Southeast Brazil, as well as to analyze the phylogenetic relationship among isolates of HTLV-1. From July to December 2017, 2,139 individuals from five Japanese associations were interviewed and submitted to blood collection. All serum samples were first tested for the presence of anti-HTLV-1/2 antibodies by ELISA and then peripheral blood from individuals with positive serological results were analyzed for the presence of HTLV-1 5’LTR proviral DNA. Partial sequencing of the 5’LTR region of HTLV-1 proviral DNA was performed by Sanger. The prevalence of HTLV-1 infection was 5.1% (CI 95%: 4.2–6.0). In the multiple logistic regression model, HTLV-1 infection was associated with age ≥ 45 years, female sex, being first and second-generation Japanese immigrants, and having sexual partners with history of blood transfusion. The phylogenetic analysis revealed that all HTLV-1 were classified as Cosmopolitan (1a) subtype. Of them, 47.8% were classified as Transcontinental (A) subgroup and 52.2% as belonging to the Japanese (B) subgroup. Although most HTLV-1-infected patients were asymptomatic (97.3%), blurred vision was associated with HTLV-1 infection. The high prevalence of HTLV-1 infection found in this studied population and especially the intra- and interfamily HTLV-1 transmission presents an urgent call for preventive and control responses of this infection in Brazil.

## Author summary

What is the prevalence of HTLV-1 among the largest world population of Japanese immigrants and their descendants outside Japan? There are approximately 1.5 million people of Japanese descent in Brazil and most of them living in São Paulo state, Southeast Brazil. The prevalence of HTLV-1 infection was 5.1% among 2,139 individuals from five Japanese associations in São Paulo. The prevalence of 5.1% (95% CI: 4.2–6.0) of HTLV-1 infection found in this study is higher than those observed in other studies conducted in HTLV-1 endemic areas and in specific groups at high risk from Brazil. Among HTLV-infected individuals, the majority (86.2%) were unaware of their HTLV diagnosis. The HTLV-1 infection was associated with the age ≥ 45 years, being the first and second generation of Japanese immigrants, and having sexual partners with a history of blood transfusion. Epidemiological data, along with molecular results also demonstrated high occurrence of similar sequences transmitted intra- and interfamily. The high prevalence of HTLV-1 infection found in this studied population presents an urgent call for preventive and control responses of this infection in Brazil.

## Introduction

Human T-cell leukemia virus type 1 (HTLV-1) has been associated with an aggressive malignant disease known as adult T-cell leukemia/lymphoma (ATL) and a chronic inflammatory neurological disease known as HTLV-1-associated myelopathy/tropical spastic paraparesis (HAM/TSP) [1]. Additionally, inflammatory disorders such as uveitis, dermatitis, arthritis, myositis, and an immune-deficient state, resulting in bronchiectasis have also been associated with this retrovirus [2].

Although most people infected by the HTLV-1 remain asymptomatic, they are viral reservoirs and maintain the HTLV-1 transmission chain, transmitting it both to their children and to their sexual partners, once HTLV-1 is horizontally transmitted through close contact with fresh blood and unprotected sexual intercourse, as well as vertically through prolonged breast feeding [3–5].

It is estimated that at least 5 to 10 million individuals are infected with HTLV-1 worldwide, but this might be underestimated [1]. HTLV-1 infection is endemic in Southern Japan [6], some parts of South America [4], sub-Saharan Africa [7], the Caribbean islands [8], part of Iran [9] and central Australia [10]. Japan has the highest prevalence for HTLV-1 infection in the world, mainly in specific areas such as the islands of Shikoku, Kyushu and Okinawa. The total number of HTLV-1 carriers in Japan was estimated to be 1.08 million with highly variable geographical distribution of HTLV-1 seroprevalence among blood donors [11]. However, Japan has been a leader in implementing several strategies to prevent new HTLV-1 infections, including antenatal HTLV-1 antibody screening, recommending seropositive mothers to refrain from breastfeeding, and also screening all blood donors for HTLV-1 infection since 1986 [12].

As far as its genotypic variability is concerned, the Cosmopolitan subtype Transcontinental subgroup (HTLV-1aA) is recognized as the most prevalent in Brazil, followed by the Japanese subgroup (HTLV-1aB) [13,14]. The existence of both these subgroups, transcontinental and Japanese, has also been reported in Japan, with an uneven distribution in endemic areas [15,16].

With the beginning of Japanese immigration to Brazil in 1908, mostly from provinces of Kumamoto, Hokkaido, Kagoshima, and Okinawa, Brazil is the country with the largest number of Japanese and their descendants outside of Japan. São Paulo state (Southeast region), followed by Paraná (South region) and Mato Grosso do Sul (Central-West region) concentrate the largest number of Japanese and their descendants in Brazil [17]. In fact, HTLV-1 infection was first identified in Brazil, in 1986, among Japanese immigrants living in Campo Grande city, Mato Grosso do Sul state [18]. Since this period, several epidemiological studies have been conducted in the Brazilian general population as well as in specific groups such as Japanese immigrants and their descendants [3,13,19–21].

Since South America has been considered as a very important focus of HTLV-1 carriers and associated diseases, correct estimates of the epidemiological and molecular aspects of the HTLV-1 infection in the city of São Paulo (the largest community of Japanese descendants outside Japan) are important to develop healthcare strategies to reduce new transmissions of HTLV-1.

## Methods

### Ethics statement

The study was approved by the Ethics Committee on Research Involving Human Subjects of the Federal University of Mato Grosso do Sul (CEP/UFMS), under protocol number 2.154.785 CAAE: 70626017.4.0000.0021. Written informed consent was obtained from all study participants prior to enrollment.

### Subjects and study design

This cross-sectional study was conducted among Japanese immigrants and their descendants living in São Paulo. After the project was disclosed to the community by means of educational lectures and distribution of flyers and leaflets with information on symptoms, prevention and diagnosis of HTLV-1/2 infection, participants were recruited from five Japanese Associations in the state of São Paulo (named A to E) between July and December 2017.

Individuals were eligible to participate if they were Japanese immigrants (born in Japan), Japanese descendants (had any genetic relationship to a Japanese immigrant) or nondescendants who had a relationship to a Japanese immigrant by marriage, living in São Paulo and able to provide written informed consent. Based on an estimated prevalence of HTLV-1 infection of 6.8% [13], a significance level of 95% (α <0.05) and an accuracy of 5%, sample size calculation, stratified by all Japanese associations, yielded 1,496 individuals. We added 20% more individuals (total of 1,795) to account for anticipated loss due to refusal to participate.

A total of 2,139 volunteer individuals agreed to participate. Informed written consent was obtained after a detailed explanation of the study at the time of blood collection from all participants or their legal guardians in case of individuals under age 18 or with a mental disability. After written informed consent, each volunteer participant was interviewed on sociodemographic characteristics, risk factors associated with HTLV infection and on their history of medical past. Right after the application of the questionnaire, blood samples were collected. In addition to an anti-HTLV test, a free medical examination was offered to all participants and non-participants by the health campaign (named Kenko Kodo). Free testing for diabetes and blood pressure were also proposed.

### Laboratorial analysis

The diagnostic criteria to define the HTLV-1 infection included an initial screening test (GOLD ELISA HTLV-I/II, REM, São Paulo, Brazil), and the repeatedly reactive samples by screening assay were checked by confirmatory analysis, using Polymerase Chain Reaction (nested PCR) or Western Blot (WB). The individuals with anti-HTLV seropositive results were contacted for further blood collection for retesting by ELISA. DNA was extracted from whole blood of HTLV seropositive samples using the QIAamp DNA Blood Mini Kit (QIAGEN, Hilden, Germany), according to manufacturer’s instructions. Nested PCR designed to amplify a 218 bp fragment of the tax gene of the both HTLV-1 and -2 was performed as previously described [13] to confirm the HTLV-1/2 infection of the seropositive samples in the screening tests. Moreover, the amplification of a 672 bp fragment of the HTLV 5’LTR region was performed on the same samples by nested PCR as previously described [13] for confirmation of the HTLV type 1 infection, specifically, and to submit its amplicons to nucleotide sequencing analysis. The tax gene region and 5’LTR analyzed in the PCR tests correspond to position 7543–7761 and position 124–796, respectively, in the ATK prototype of the Japanese HTLV-1aB strain.

The 5’LTR amplicons were purified using PureLink Quick PCR Purification Kit (Invitrogen, Lithuania), according to the manufacturer’s instructions. The fragments were sequenced using BigDye Terminator Cycle Sequencing Ready Reaction Kit and ABI 1373 (Applied Biosystems, Foster City, CA, USA) using Sanger’s method. The nucleotide sequences were edited in DNASTAR Lasergene software and aligned with published HTLV-1 sequences from various geographic regions using Mega X software [22]. These published references sequences representing the HTLV-1 subtypes a-e and worldwide sequences, including strains from distinct regions of Brazil, were selected and downloaded from the GenBank database. Maximum Likelihood (ML) phylogenetic tree of non-identical LTR sequences was constructed with the PhyML 3.0 program using an online web server [23]. The Smart Model Selection recommended the TN93+G+F nucleotide substitution model to be used in the ML [24]. The heuristic tree search was performed using the Subtree-Pruning-Regrafting branch-swapping algorithm, and the branch support was calculated with the approximate likelihood-ratio (aLRT) SH-like test [25]. The ML tree was visualized online using the Interactive Tree Of Life (iTOL) [26]. Overall mean distance and pairwise distances were conducted using the Maximum Composite Likelihood model [27]. All ambiguous positions were removed for each sequence pair. Evolutionary analyses were conducted in MEGA X [22].

Regarding family pedigree, out of 109 HTLV-1 infected individuals, 12 (OKSP1400, OKSP620, OKSP106, OKSP123, OKSP763, OKSP640, OKSP17, OKSP1608, OKSP1490, OKSP1698, OKSP1812 and OKSP1497) became the index cases and 53 of their relatives who agreed to participate of this study, including mother, father, wives, husbands, sisters, brothers-in-law, sisters-in-law, children, nephews and nieces, were investigated.

Being aware of being HTLV-infected was defined by self-report; those who had never been HTLV tested or who did not know their HTLV status were defined as being unaware of being HTLV-infected.

### Data analysis

Data was entered into the Research Electronic Data Capture (REDCap) online database. Data analysis was performed in the statistical software Stata SE, version 13 (StataCorp LP, College Station, USA). This study used the chi-square test (χ^2^) to assess differences between proportions. Prevalence rate of HTLV-1 infection and 95% confidence interval (CI) were calculated. Bivariate analysis was performed to verify potential predictors of exposure to HTLV-1/2 infection (anti-HTLV-1/2 positive). Variables that presented a p-value < 0.20 were included in multiple linear regression. The selection of variables for the final model was performed stepwise, according to the number of events per variable (EPV). Hosmer-Lemeshow test was used to assess goodness-of-fit, choosing the best regression equation. P-values <0.05 were considered statistically significant.

## Results

### Characteristics of the studied population

A total of 2,139 participants were enrolled in this study. Median age of participants was 56 years, ranging from 6 to 92 years. As shown in Table 1, the majority (68.6%) had 45 years of age or older. Most of the studied population was female (59.8%). In relation to formal education, 72.2% had more than 9 years of study. A monthly household income higher than about 857.00 USD (based on national minimum wage at the time converted to dollar) was reported by 65.0% of the participants. Regarding marital status, 74.5% had or had had stable relationships (married, divorced or widowed). The distribution of the studied population was similar by Japanese association, with participation of 33.4%, 14.2%, 20.6%, 16.2% and 15.6% individuals in A, B, C, D and E associations, respectively. Of total, 15% were born in different regions of Japan, 79% were from São Paulo State and a minority (6.1%) was born in other states of Brazil or other countries. History of living in Japan was reported by 38.7% of participants.

**Table 1:** Characteristics of Japanese immigrants and their descendants living in São Paulo (N = 2,139).

Characteristics	N^a^	%
**Age (years)**		
<45	672	31.4
≥45	1,467	68.6
**Sex**		
Male	861	40.2
Female	1,278	59.8
**Formal education (years)**		
Illiterate	18	0.8
1–9	564	26.4
10–12	591	27.6
>12	953	44.6
Monthly household income*		
<1	123	6.2
1–3	569	28.8
>3	1285	65.0
**Marital status**		
Single	546	25.5
Married, divorced or widowed	1,593	74.5
**Recruitment site**		
Association A	715	33.4
Association B	303	14.2
Association C	440	20.6
Association D	346	16.2
Association E	335	15.6
**Family heritage**		
Japanese immigrant	320	15.0
Japanese son	1011	47.3
Grandson and great-grandson of Japanese immigrant	753	35.2
Non-Japanese descendant	55	2.5
**Okinawan Descendant**		
No	266	12.44
Yes	1873	87.56
**History of residence in Japan**		
No	1311	61.3
Yes	828	38.7
**Blood transfusion**		
No	1,994	93.2
Yes	145	6.8
**Blood transfusion year**		
After 1993	68	52.3
Before 1993	62	47.7
**Surgery**		
No	738	34.5
Yes	1,401	65.5
**Shared sharp objects**		
No	1,272	59.5
Yes	867	40.5
**Accidental contact with blood of others**		
No	2,073	96.9
Yes	66	3.1
**Regular use of condoms**		
No	1,576	83.7
Yes	308	16.3
**Partner’s blood transfusion history**		
No	1,480	94.1
Yes	93	5.9

* National minimum wage: during the study period, one minimum wage was approximately R$ 937.00 BRL (U$ 285.00 USD).

^a^The total represents the number of individuals who answered the question

Some risk behaviors were less reported in this population, such as history of tattooing (7.2%), piercing (4.1%), having been in prison (0.3%), injection drug use (0.1%) and multiple sexual partners (4.2%). Nearly 7% of the participants reported having received a blood transfusion, and of these, almost half was before 1993, when compulsory HTLV-1 screening was implemented for Brazilian blood donors.

### Prevalence of HTLV-1 infection and risk factor analysis

**Fig 1:**
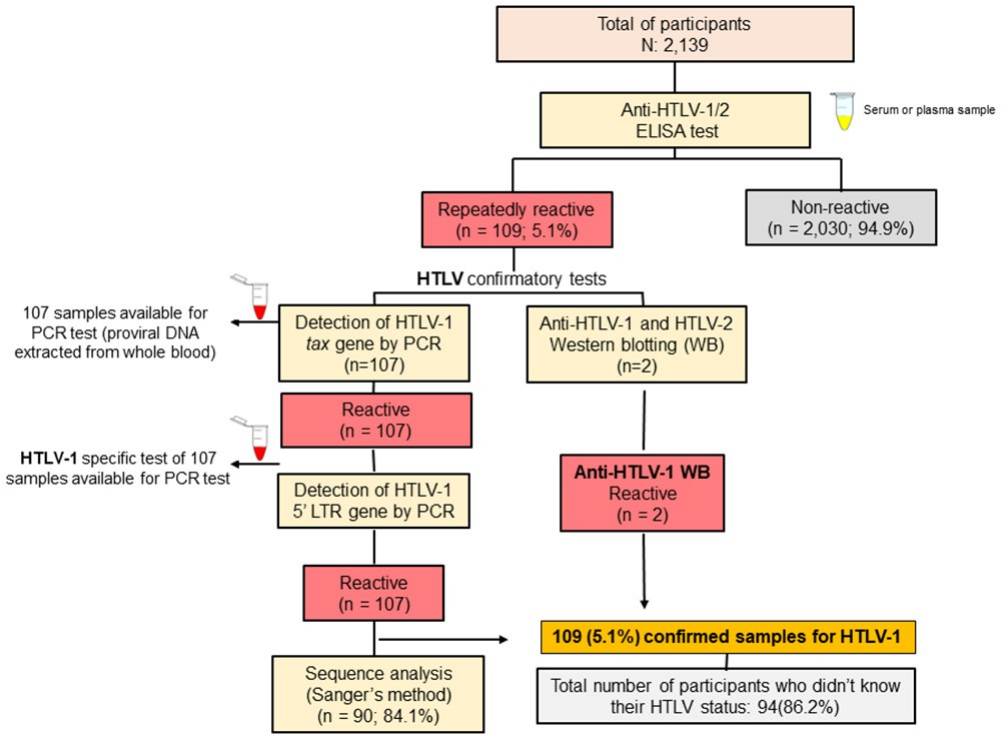
Flow chart of the screening and confirmation of HTLV-1 infection.

Out of 2,139 participants, 109 were repeatedly reactive for anti-HTLV-1/2 antibodies by ELISA. Of the 109 positive samples in the screening tests, 107 amplified the HTLV *Tax* gene region by nested PCR, thus confirming the HTLV infection. The same 107 samples also amplified the HTLV-1 5`LTR region, thus confirming the HTLV-1 infection. The two positive samples remaining in the screening tests were confirmed for the HTLV-1 infection by Western Blot, since there was no whole blood sample from the respective individuals to perform the DNA extraction for the nested PCR. Therefore, considering that a total of 109 individuals were confirmed for infection by the HTLV-1, a prevalence of 5.1% (CI 95%: 4.2–6.0) of HTLV-1 infection was thus found (Fig 1). Among the 109 individuals who tested positive for HTLV, only 15 (13.8) knew themselves to be HTLV-infected. Thus, 94 (86.2%) were unaware of their diagnosis.

**Fig 2:**
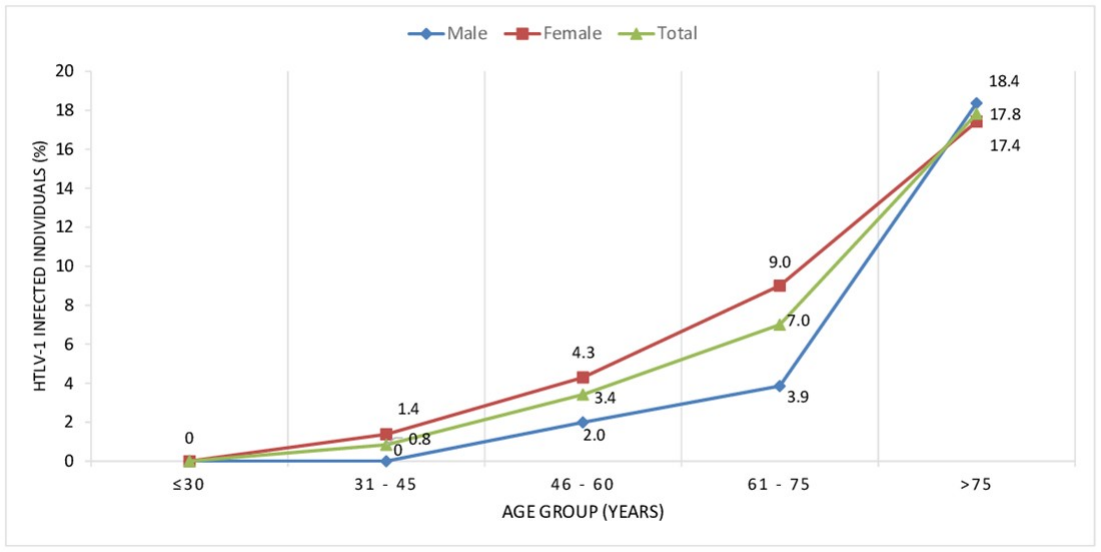
Changes according to age group and sex in HTLV-1 infected individuals.

Table 2 presents the bivariate and multivariate logistic regression analyses results for risk factors. In the multiple logistic regression model HTLV-1 infection was associated with age ≥45 years (p = 0.025), female sex (p = 0.040) (Fig 2), being first and second-generation Japanese immigrants (p = 0.002 and p = 0.017, respectively) and with report of having sexual partners with history of blood transfusion (p = 0.014). There was a difference in the prevalence of HTLV-1 infection among the five associations included in the study, ranging from 3.3% in Association A to 8.9%(CI 95%: 6.3–12.5) in Association E (p<0.001). There was no difference in the prevalence of HTLV-1 infection between Okinawan descendants and descendants from other regions of Japan (unspecified) and non-Japanese descendants.

**Table 2:** Factors associated with HTLV-1 infection among Japanese immigrants and their descendants in São Paulo (N = 2,139).

Variables	HTLV-1 prevalence^a^ N (%)		OR	*p*-value	Adjusted OR^b^ (95%CI)	*p*-value^b^
**Age (years)**						
< 45	3/672	0.5	1		1	
≥45	106/1467	7.2	17.37	<0.001*	5.68	0.025*
**Sex**						
Male	32/861	3.7	1		1	
Female	77/1278	6.0	1.67	0.018*	1.67	0.040*
**Marital status**						
Single	5/546	0.9			1	
Married, divorced or widowed	104/1593	6.5	7.56	<0.001*	2.67	0.357
**Recruitment site**						
Association A	24/715	3.4	1			
Association B	13/303	4.3	1.29	0.468		
Association C	21/440	4.8	1.44	0.229		
Association D	21/346	6.1	1.86	0.043		
Association E	30/335	9.0	2.83	0.000*		
**Family heritage**						
Grandson/great-grandson of Japanese	11/753	1.46	1		1	
Japanese son	62/1011	6.13	4.41	<0.001*	2.74	0.017*
Japonese immigrant	35/320	10.94	8.28	<0.001*	4.024	0.002*
**Okinawan descendant**						
No	7/266	2.63	1		1	
Yes	102/1873	5.45	2.13	0.056*	2.06	0.128
**History of residence in Japan**						
No	63/1311	4.81	1			
Yes	46/828	5.56	1.17	0.443		
**Natural birth**						
No	5/408	1.23	1		1	
Yes	101/1592	6.34	5.46	<0.001*	0.96	0.937
**History of breastfeeding**						
No	1/56	1.79	1			
Yes	98/1905	5.14	2.98	0.281		
**Blood transfusion**						
No	96/1994	4.81	1		1	
Yes	13/145	8.97	1.95	0.031*	0.97	0.943
**Surgery**						
No	28/738	3.79	1		1	
Yes	81/1401	5.78	1.56	0.049*	0.73	0.217
**Tattooing**						
No	103/1985	5.19	1			
Yes	6/154	3.90	0.74	0.484		
**Shared sharp objects**						
No	66/1272	5.19	1			
Yes	43/867	4.96	0.95	0.813		
**Accidental contact with blood of others**						
No	104/2073	5.02	1			
Yes	5/66	7.58	1.55	0.356		
**Ever homossexual contact**						
No	107/2031	5.27	1			
Yes	2/46	4.35	0.82	0.782		
**Sexually transmitted infection (STI)**						
No	101/1929	5.24	1			
Yes	8/149	5.37	1.03	0.944		
**Condom use**						
Yes	7/308	2.27	1		1	
No	98/1576	6.22	2.85	0.008*	1.48	0.413
**Number of sex partners/year**						
≤1	104/1772	5.87	1			
>1	2/91	2.20	0.36	0.158		
**Partner’s blood transfusion history**						
No	81/1480	5.47	1		1	
Yes	12/93	12.90	2.56	0.004*	2.31	0.014*
**Partner with STI**						
No	90/1563	5.76	1			
Yes	4/47	8.51	1.52	0.431		

95% CI: 95% Confidence interval; OR: Odds ratio;

^a^The total represents the number of individuals who answered the question.

^b^Adjusted for age and family heritage.

All anti-HTLV-1 infected individuals and their relatives and/or contacts were recalled for a new blood collection to confirm the result. On this occasion, all anti-HTLV-1 infected individuals were submitted to the first clinical screening by a neurologist of our group and later referred to a hematology specialist in the HTLV outpatient clinic at Hospital das Clínicas, University of São Paulo, located in São Paulo city. Of all those infected, 61.5% have attended the clinic to date and the majority remain asymptomatic (97.3%), however one individual presented the chronic form of ATL and two of them depicted the possible factor qualification for HAM/TSP (HAM-PS). In addition, participants who reported blurred vision had 2.1-fold greater chance of being HTLV-1 positive compared with those without this symptom (Table 3).

**Table 3:** Symptoms associated with HTLV-1 infection among Japanese immigrants and their descendants in São Paulo (n = 2,139).

Variables	HTLV-1 prevalence^a^ N (%)		OR	*p*-value	Adjusted OR^b^ (95%CI)	*p*-value
**Age (years)**						
< 45	3/672	0.5	1		1	
≥45	106/1467	7.2	17.37	<0.001*	14.71	<0.001*
**Sex**						
Male	32/861	3.7	1		1	
Female	77/1278	6.0	1.67	0.018*	1.53	0.059
**Difficulty in urinating**						
No	92/1884	4.9	1			
Yes	17/255	6.7	1.39	0.226		
**Incontinence**						
No	89/1922	4.6	1		1	
Yes	20/214	9.2	2.09	0.004	1.20	0.502
**Erectile dysfunction**						
No	26/757	3.4	1			
Yes	6/101	5.9	1.78	0.218		
**Joint pain**						
No	70/1585	4.4	1		1	
Yes	39/554	7.0	1.64	0.017	0.97	0.891
**Arthritis**						
No	93/1914	4.9	1		1	
Yes	16/225	7.1	1.50	0.149	0.81	0.480
**Dry mouth and eyes**						
No	80/1743	4.6	1		1	
Yes	29/396	7.3	1.64	0.027	1.07	0.772
**Dermatitis**						
No	94/1778	5.3	1			
Yes	15/360	4.2	0.78	0.374		
**Blurred vision**						
No	76/1819	4.2	1		1	
Yes	33/320	10.3	2.64	<0.001	1.99	0.004*
**Walking difficulty**						
No	93/1970	4.7	1			
Yes	16/169	9.5	2.11	0.008		
**Fast walking difficulty**						
No	85/1895	4.5	1		1	
Yes	24/244	9.8	2.32	<0.001	1.29	0.347
**Running difficulty**						
No	81/1787	4.5	1			
Yes	28/352	7.9	1.82	0.008		
**Spasms**						
No	95/1955	4.9	1		1	
Yes	14/184	7.6	1.61	0.108	1.23	0.505
**Clonus**						
No	107/2111	5.1	1			
Yes	2/28	7.1	1.44	0.622		
**Frequent falls**						
No	103/2093	4.9	1		1	
Yes	6/46	13.0	2.90	0.018	1.27	0.618
**Numbness of the lower limbs**						
No	100/1952	5.1	1			
Yes	9/187	4.8	0.94	0.854		
**Walk Help**						
No	106/2114	5.0	1		1	
Yes	3/25	12.0	2.58	0.128	1.21	0.770
**Legs paralysis**						
No	107/2125	5.0	1			
Yes	2/14	14.3	3.14	0.137		
**Lymphoma**						
No	108/2127	5.1	1			
Yes	1/12	8.3	1.70	0.613		

95% CI: 95% Confidence interval; OR: Odds ratio;

^a^The total represents the number of individuals who answered the question.

^b^Adjusted for age and sex.

### Molecular epidemiology of HTLV-1 infection

**Fig 3:**
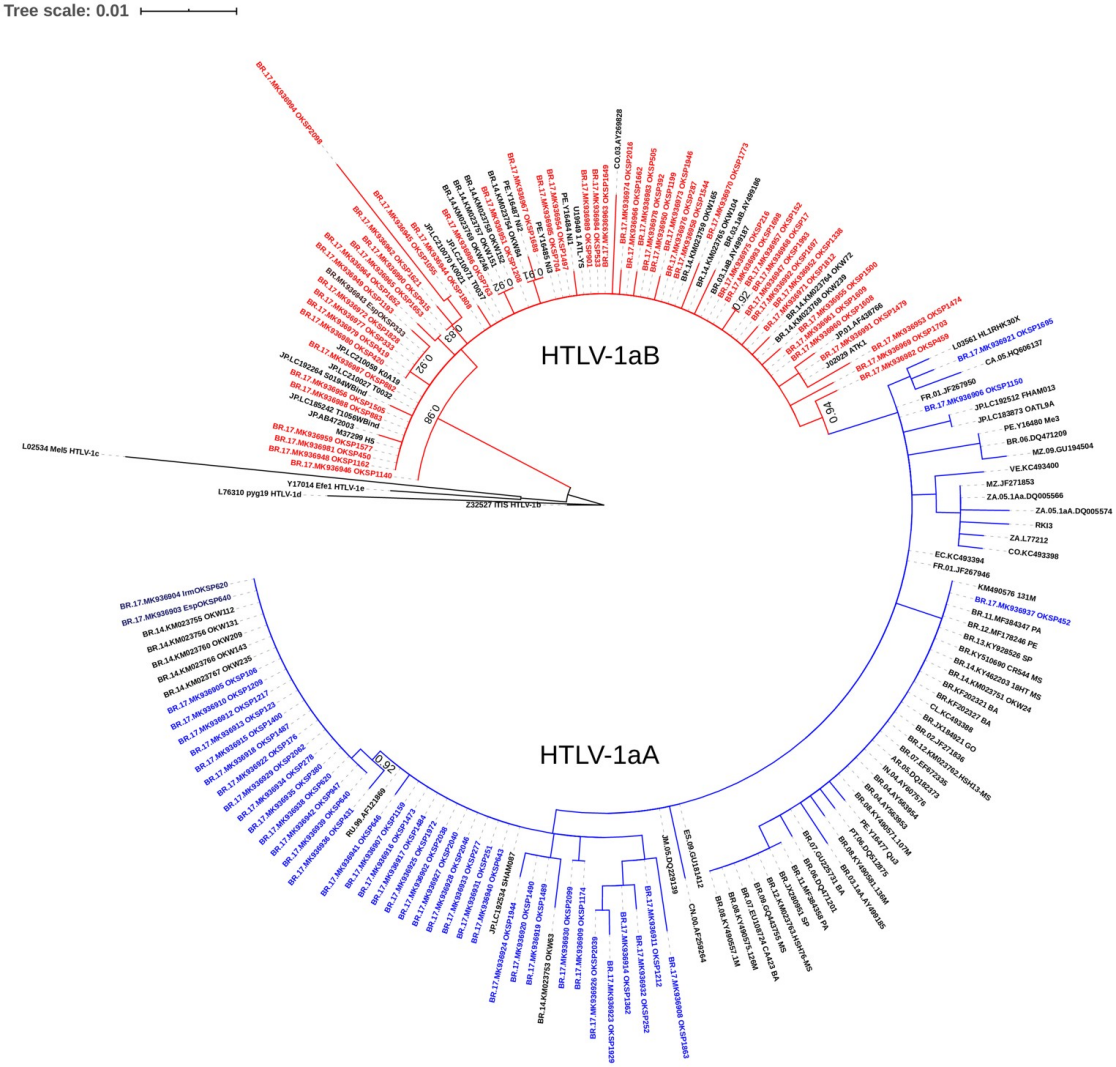
Phylogenetic tree based on partial 5’LTR region (400bp) of HTLV-1 constructed by Maximum Likelihood (ML) method of 90 HTLV-1 proviral DNA (OKSP) and 93 sequences available in GenBank. aLRT values are represented only if higher than 0.90. The new HTLV-1 isolates belonging to the Transcontinental (A) subgroup and to the Japanese (B) subgroup were highlighted in blue and red, respectively.

The HTLV-1 proviral DNA was detected by amplification of the 5’LTR region by nested PCR in 98.2% (107/109) of anti-HTLV-1 positive samples. The remaining two positive samples were confirmed only by Western Blot (MP Diagnostics HTLV BLOT 2.4–Singapore), due to difficulty of venipuncture during the collection of new samples. Out of 107 samples with amplified LTR, 90 (84.1%) were successfully sequenced. The phylogenetic analysis of the HTLV-1 5’LTR region revealed that all isolates were classified as Cosmopolitan (1a) subtype. Of these, 47.8% HTLV-1 isolates were classified as belonging to the Transcontinental (A) subgroup and 52.2% to the Japanese (B) subgroup (Fig 3). The HTLV-1 nucleotide sequences of amplified positive samples are available in GenBank and assigned NCBI accession numbers: MK936902-MK936994. Distance estimation of HTLV-1 nucleotide sequences from studied isolates showed that the overall mean distance among sequences classified as 1aA was 0.5%, ranging from 0.0% to 1.9%. The overall genetic distance observed on HTLV-1 viral isolates classified as 1aB was 0.5%, ranging from 0.0% to 2.5%. Pairwise distances were also calculated to estimate distances among three clusters with closely related sequences from our study (aLRT≥0.9) and the pairwise genetic distances were 0.0%. Of these three clusters, two were composed by couples (OKSP419 and OKSP420, EspOKSP333 and OKSP333, OKSP152 and OKSP17). The other cluster was composed by an isolate from our study (OKSP1688) with an isolate from a Nikkei individual from Peru (Ni2) and with an Okinawan descendant from Campo Grande county (State of Mato Grosso do Sul) (OKW84).

**Fig 4:**
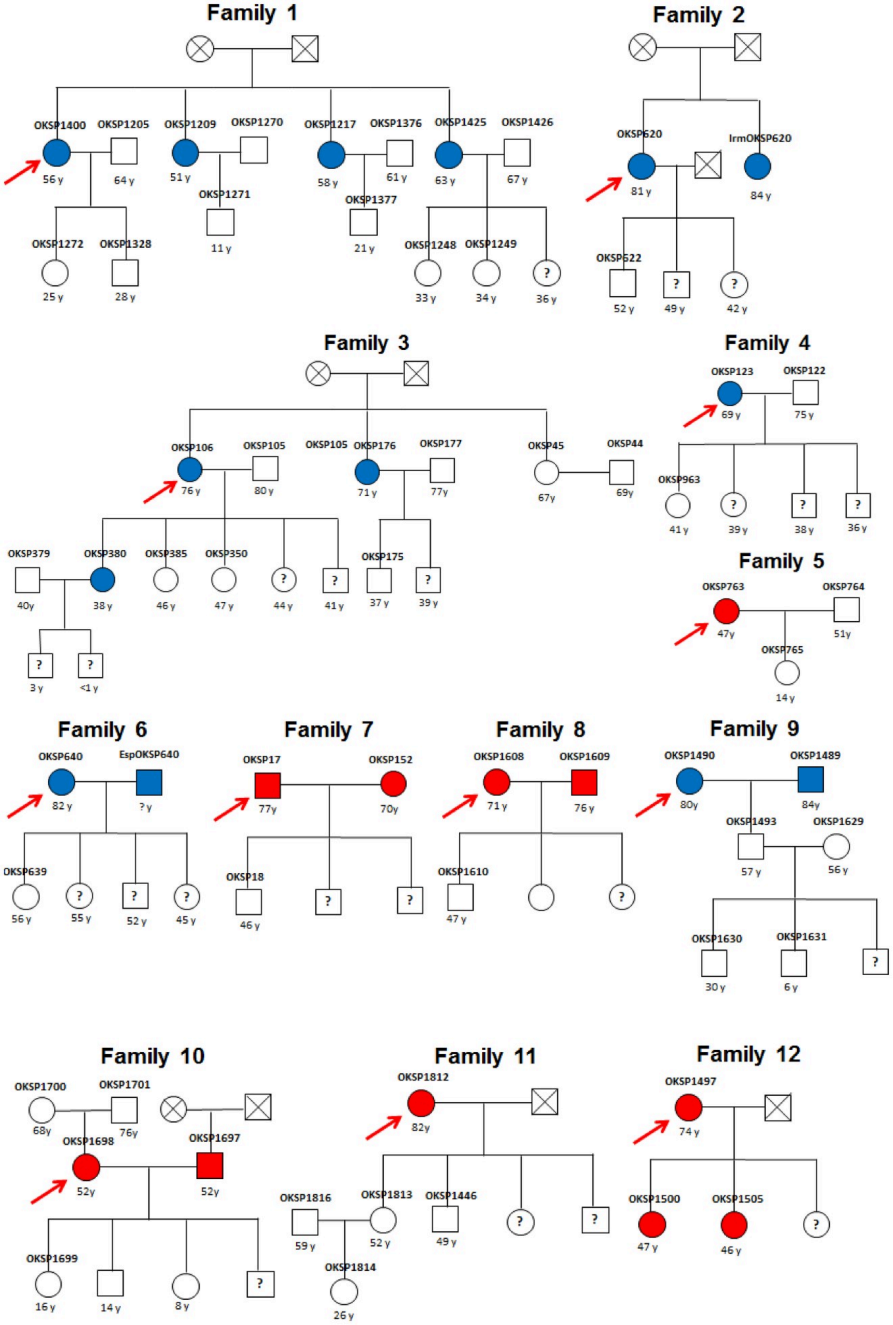
Pedigree of the twelve families of index cases under study. Square, male; Circle, female; Blue full square/circle, HTLV-1aA-infected individual; Red full square/circle, HTLV-1aB-infected individual; Empty square/circle, uninfected individual; Square/circle with question mark, unknown HTLV-1 status; Square/circle with a cross inside, deceased. Index case.

Fig 4 shows the family pedigrees of the index cases (one per family) under study. Thirteen out the 53 (24.5%) relatives were anti-HTLV-1 positive. In the majority of these 12 families (75%), at least two members tested anti-HTLV-1 positive. Genetic distance between viral isolates of each family group was 0.0%. The exception was OKSP1425 whose viral isolate could not be sequenced. It is important to note that in family 1, the viral isolate sequenced from the index case (OKSP1400) showed 100% identity with two of her sisters’ (OKSP1209 and OKSP1217). All of them reported having been breastfed. None of their husbands were HTLV-1-infected. All of the positive participants’ offspring were tested negative for anti-HTLV-1. In family 3, the sister (OKSP45) of the index case (OKSP106) was anti-HTLV negative and was the only sister that was not breastfed. Although all tested OKSP106 offspring were breastfed for more than 6 months, only one (OKSP380) was HTLV-1-infected and presented 100% identity with her mother, suggesting vertical transmission. There was no evidence of sexual transmission from female to male in families 4 and 5. In contrast, in families 6, 7, 8, 9 and 10, genetic distance between viral isolates of index cases and their couples showed 100% identity, evidencing sexual transmission. Although their index cases’ tested children were breastfed for 6 months or more, they were not HTLV-1-infected. It is worth mentioning that in spite of the viral isolates from HTLV-1 infected members of family 12 (mother and two daughters; OKSP1497, OKSP1500 and OKSP1505, respectively) showing 100% identity, the index case (OKSP1497) reported not having breastfed any of her daughters, suggesting transmission during pregnancy or childbirth.

## Discussion

This large cross-sectional survey was conducted to investigate HTLV-1 infection in Brazil among the world’s largest Japanese population outside of Japan. There are approximately 1.5 million people of Japanese descent in Brazil and most of them living in São Paulo state [17]. The prevalence of 5.1% (95% CI: 4.2–6.0) of HTLV-1 infection found in this study is higher than those observed in other studies conducted in different regions of the country considering HTLV-1 endemic áreas, including Salvador, the city with the highest HTLV-1 prevalence (1.48%) in Brazil [28–31], and higher than those conducted among specific risk groups, such as men who have sex with men, people who used illicit drugs, HIV-infected persons, and commercial sex workers, with prevalence rates varying from 0.7% to 3.0% [32–35].

This high rate (5.1%) found in the present study is similar to that observed in previous study involving Japanese community of Campo Grande, Mato Grosso do Sul State (6.8%) [13] probably because the majority of subjects in both studies was descendant of Okinawans as well as of people from other HTLV-1-endemic regions in Japan. The presence of HTLV-1 infecting Japanese immigrants residing in Tomé-Açu county (State of Pará) was also described [21]. These findings could be due to the introduction of HTLV-1 in South America through Japanese immigration from the early 20^th^ century [36]. Also, this study has highlighted that a very high percentage of HTLV-infected were unaware of their infection and they must be promptly linked and retained in health-care to benefit their health and reduce transmission to others.

Similarly to findings from previous studies, the prevalence of HTLV-1 infection in our cohort was higher among females than in males (p = 0.04). Although vertical transmission is not sex-biased, sexual transmission is female-biased, which explains why females overall have a higher HTLV-1 carrier rate than males [37]. Moreover, there are more lymphocytes in semen than in vaginal secretions, which may explain the more efficient transmission from male to female [38]. Our group has previously shown that both vertical and sexual transmissions occur among Japanese immigrants and their descendants in Brazil [3]. This epidemiological finding, along with molecular results demonstrates that sexual transmission of HTLV-1 is present in this population in addition to vertical transmission.

Increasing age was associated with HTLV-1 infection in our cohort, probably due to prolonged exposure to HTLV-1 infection, which is a well-known characteristic and observed in other populations in previous studies [11,13,39–41]. In Japan, the decline of HTLV-1 seroprevalence in younger generations may be caused due to the effects of the successful HTLV-1 screening program for pregnant women and the shortening of the breastfeeding period in recent decades [11]. In Brazil, HTLV-1 antenatal screening was implemented in a few cities, such as Campo Grande, by the Program for the Protection of Pregnant Women in the State of Mato Grosso do Sul and in Salvador, the Brazilian city with the highest HTLV-1 prevalence [42]. However, in most Brazilian cities, HTLV-1 antenatal screening is not routinely performed by the national health system (SUS) [43]. We thus assume the increased prevalence in older individuals may be due to the cumulative risk of HTLV-1 infection over the lifetime, including prolonged exposure to an infected sexual partner, prolonged breastfeeding and also the closer kinship to Japanese immigrants from endemic areas. In fact, Satake et al. (2016) highlighted that older couples usually do not use condom and that ageing leads to the senescence and subsequent deterioration of defense mechanisms against infection [6]. The same hypothesis may also apply to justify the higher prevalence of HTLV-1 infection found in participants of our cohort who belonged to the first and second generation of Japanese immigrants. Perhaps if preventive and control measures had been taken in the past, most infections among first and second generation individuals could have been avoided. In fact, the low prevalence rate of HTLV-1 infection among newer generations may be due to the shortening of the breastfeeding period, associated with the growing participation of women in the labor market, which can lead to changes in breastfeeding patterns [44].

To reinforce these findings, when male and female were analyzed separately, HTLV-1 infection was independently associated with being a first or second (p = 0.01) generation immigrant for both males (p = 0.002 and p = 0.01, respectively) and females (p = 0.004 and 0.027, respectively). Moreover, for female participants having partners with a history of receiving blood transfusions was also associated with HTLV-1 infection (p = 0.033).

A higher prevalence of HTLV infection was found in the population coming from Association E (8.9%). This fact can be explained by the higher proportion of first Japanese generation participants in this association when compared to the others (p<0.001). HTLV-1 infection is endemic in Japan, particularly clustered on the islands of Kyushu and Okinawa, in southwestern Japan [11]. Although it is well-known that Okinawa island is an endemic area, no association between HTLV-1 infection and being an Okinawan descendant in this study was found, probably because most of HTLV-1-infected non-Okinawan descendants were related to Okinawan descendants or were from other regions of Japan where prevalence of HTLV-1 infection is also high.

As previously mentioned, having a partner with a history of receiving blood transfusion history was independently associated with HTLV-1 infection in our cohort. However, we should highlight that only three of the nine HTLV-1-positive women who reported so, informed their partners received blood transfusions before 1993. Of these three women, two were widows and therefore, we did not have access to results of their partners’ HTLV-1 antibody screening. The other one was married to an HTLV-1-non-infected individual. Among the other six women, five reported that their husbands’ blood transfusions occurred after 1993 and one did not know when it happened. Of these six women, 4 were married to subjects who participated in this study and of these, 3 were anti-HTLV-1 positive. Serological screening for HTLV-1/2 became mandatory in blood banks in Brazil in 1993, effectively reducing the risk of HTLV-1 transmission by transfusion [45].

The lack of association of HTLV-1 infection with STI differs from other studies conducted in Brazil [28,33]. This result highlight the importance of mother-to-child transmission (MTCT) among this population. Evidence of vertical transmission as an important HTLV-1 route of HTLV-1 has been reported in previous studies conducted in Brazil and Japan [3,13,43,46]. Together, these findings highlight the importance of prenatal care and early maternal diagnosis to prevent HTLV transmission from mother to child.

According to Kamoi and Mochizuki (2012), all patients with initial diagnosis of HTLV-1 infection should be strictly screened for ocular symptoms [47]. Although blurred vision was associated with HTLV-1 infection in this study, other symptoms, as well as eye pain/burning, itching, and foreign body sensation, are also the most common clinical manifestations of HTLV-1-associated uveitis. Other reported symptoms, such as incontinence, arthritis, joint pain, dry mouth and eyes, dermatitis, difficulty in walking and running and frequent falls, were not statistically associated with HTLV-1 infection in our cohort. It is important to note that the majority of the studied population was 45 years old or older (68.6%) and that these non-specific symptoms are usually reported by older people.

The characteristic clinical picture of the chronic spastic paraparesis is not always easily found, sometimes the patient depicts symptoms or signals that are incipient indicators of HAM/TSP [48]. In Brazil, De Castro-Costa et al. (2006) elaborated new criteria that proposed three levels of certainty for the diagnostic definition of HAM/TSP. The HTLV-1 symptomatic carriers were considered possible HAM/TSP (HAM-PS) by presenting pyramidal weakness, reflex alteration, and urinary urgency [49]. It is possible to explain the few cases of ATL in this population: Most of the ATL cases may die early in life, without diagnosis. Another possibility is that the Brazilian genetic background may not susceptible for ATL development, both hypotheses are necessary to be studied in this population [50].

The phylogenetic analysis revealed that all HTLV-1 belong to the Cosmopolitan (1a) subtype and 47.8% are from the Transcontinental (A) subgroup and 52.2% to the Japanese (B) subgroup. These two subgroups occur in Japan, with the prevalence of the Japanese [14–16]. In Brazil, HTLV-1aB is associated with Japanese or descendant individuals [13,21,51]. Epidemiological data, along with distance estimation and pairwise distances analysis demonstrated that HTLV-1 from this population are highly conserved providing evidence on the transmission within family clusters corresponding to Japanese immigrants and their descendants in São Paulo state.

Some limitations found in this study should be considered. Firstly, recall bias may have impaired the reliability of information provided by study participants about risk behaviors, such as about having been breastfed in childhood. But it is noteworthy that cross-breastfeeding was a frequent past habit, reported to occur on ships that brought the first Japanese immigrants, according to our interviewees. Moreover, the number of symptomatic HTLV-1 carriers may have been underestimated, since difficulty in walking, the main clinical manifestation of the HAM/TSP, may have led to the non-participation of some subjects in the study. Despite these limitations, our study, having a large sample size, is representative of this population group and provides important novel insights about HTLV-1 infection in Brazil, where the largest population of Japanese and their descendants outside Japan live.

In conclusion, our study demonstrates that the prevalence of HTLV-1 infection is high among Japanese immigrants and their descendants in the state of São Paulo, where the largest number of Japanese and their descendants outside Japan live. Epidemiological data, along with molecular results also demonstrated high occurrence of similar sequences transmitted intra- and interfamily. Therefore, our results on this neglected disease highlight the urgent need of preventive strategies to decrease spread and transmission of this virus in this area of Brazil, including i) effective HTLV testing strategies and routine systematic HTLV-1/2 antenatal screening and counseling of Brazilian pregnant women by the national health system (Sistema Único de Saúde, SUS); ii) health educational programs to avoid sexual and other intrafamily horizontal transmissions; iii) systematic counseling of HTLV-1-infected individuals to reduce risk of viral transmission.
